# Avian hepatitis E virus is widespread among chickens in Poland and belongs to genotype 2

**DOI:** 10.1007/s00705-018-4089-y

**Published:** 2018-11-03

**Authors:** Anna Karolina Matczuk, Katarzyna Ćwiek, Alina Wieliczko

**Affiliations:** 1 Department of Pathology, Division of Microbiology, Faculty of Veterinary Medicine, Wrocław University of Environmental and Life Sciences, Norwida 31, 51-375 Wrocław, Poland; 2 Department of Epizootiology with Clinic of Birds and Exotic Animals, Faculty of Veterinary Medicine, Wrocław University of Environmental and Life Sciences, pl. Grunwaldzki 45, 50-366 Wrocław, Poland

## Abstract

Big liver and spleen disease, caused by avian hepatitis E virus, has been reported in Poland, but the prevalence of the virus has not yet been investigated. In this study, 1034 serum samples from 57 breeder broiler and laying hen flocks were screened for the presence of anti-aHEV antibodies. In a random serology study, 56.1% of flocks were positive. Seroprevalence was higher in laying hen flocks than in broiler breeder flocks. Phylogenetic analysis of partial ORF1 and ORF2 sequences revealed that all Polish isolates belonged to genotype 2. This is the first time this genotype has been detected in Central Europe.

**Electronic supplementary material:**

The online version of this article (10.1007/s00705-018-4089-y) contains supplementary material, which is available to authorized users.

Avian hepatitis E virus (aHEV) is a non-enveloped, single-stranded RNA virus belonging to the genus *Orthohepevirus*, family *Hepeviridae.* Four species have been defined: *Orthohepevirus A*, *B*, *C* and *D*. *Orthohepevirus B* includes avian HEV isolates from chickens, while the other three species include HEV isolates from mammals [1]. The genome of aHEV is 6.6kb long and contains three open reading frames (ORFs) [2]. ORF1 encodes a non-structural polyprotein that contains cysteine protease, methyltransferase and RNA helicase domains. The gene encoding the viral capsid protein (ORF2) is overlapped by ORF3, which encodes a cytoskeleton-associated phosphoprotein [2, 3]. Members of the species *Orthohepevirus B* can be divided in four separate genotypes [4, 5].

Hepatitis E virus is associated with big liver and spleen (BLS) disease, also described as hepatitis-splenomegaly syndrome (HSS). It was first reported in chickens in Australia in the 1980s [3]. The first cases in Europe were reported in Italy and Hungary in 2004 and 2005, respectively [6, 7]. In Poland, veterinarians have been observing cases of BLS disease since at least 2007, although the first partial ORF sequences of Polish aHEV isolates were described and submitted to GenBank in 2010 [4]. Genotype 1 has been identified in Australia and Korea, genotype 2 is present in North America and Spain, genotype 3 is present in Europe, China and, to some extent, in North America, while a novel putative genotype 4 has been detected in Hungary and Taiwan [8–10]. Chickens affected by BLS typically have enlarged livers and spleens and blood-stained fluid in the abdomen, accompanied by a drop in egg production (10%-40%) and elevated mortality rates (1%-4%) [11]. Both anti-aHEV antibodies and viral RNA have been detected in healthy chicken flocks, suggesting that aHEV mainly causes subclinical infections [10, 12, 13].

Because the epidemiological status of aHEV has not yet been assessed in Poland, the aim of this study was to evaluate the seroprevalence of aHEV in Polish chicken flocks. Moreover, aHEV genetic material was isolated from the seropositive flocks, and the nucleotide sequences present in these flocks were subjected to phylogenetic analysis.

Serological studies were carried out using 1034 serum samples from 57 flocks (46 breeder broiler flocks and 11 laying hen flocks) collected between 2015 and 2017 in western and southern Poland. No clinical data were obtained for those flocks. From each flock, 15 to 23 sera were collected for further study. Serum samples were stored at -20 °C until analysis. The anti-aHEV antibody titer in chicken sera was measured using a commercial ELISA kit (Big Liver and Spleen Disease Antibody Test Kit, BioCheck, The Netherlands) according to the manufacturer’s protocol. To investigate the genotype of aHEV, the internal organs of birds (livers and spleens) were collected from farms where we had earlier detected seropositive birds and from other flocks in which a drop in egg production or anatomopathological changes in spleen and liver had been observed. From May 2017 to February 2018, 65 flocks with different production profiles were examined. Viral RNA was isolated from five pooled livers and five pooled spleens from each flock using a Total RNA Mini Plus Kit (A&A Biotechnology, Poland). RNA was transcribed into DNA using a Maxima H Minus First Strand cDNA Synthesis Kit (Thermo, Poland), followed by nested PCR reactions specific for ORF1 fragments (helicase gene) and ORF2 (capsid gene) described previously [12]. PCR products of the correct size (386 bp and 242 bp, respectively) were excised from agarose gels, purified using Gel-Out (A&A Biotechnology, Poland), and sequenced in both directions (Genomed, Warsaw). If the RT-PCR was positive for both spleen and liver samples, only the PCR product from the liver sample for this flock was subjected to sequencing. Phylogenetic analysis was performed based on a nucleotide sequence comparison of the ORF1 and ORF2 gene fragments from the aHEV strains isolated in Poland and nucleotide sequence of other strains available in the GenBank database, using the MEGA 7.0 and BLASTn programs. Phylogenetic trees were generated by the NJ method (Fig. 1a) and ML method (Fig. 1b) as implemented in the MEGA 7.0 software. The robustness of the trees was evaluated by bootstrapping of multiple sequence alignments (1000 sets).

**Fig. 1:**
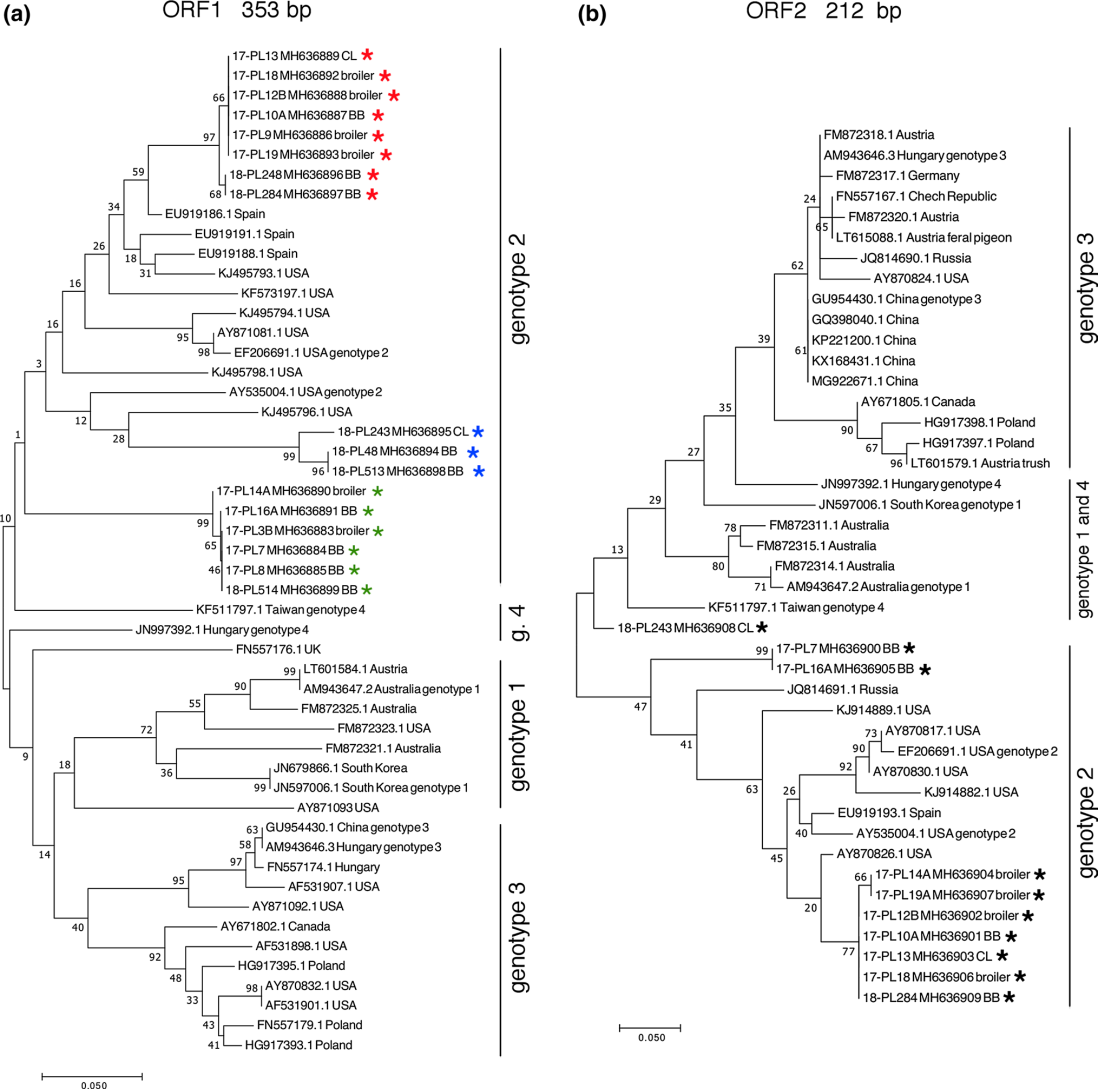
Phylogenetic analysis based on partial ORF1 (helicase) (**a**) and partial ORF2 (capsid) (**b**) gene sequences of aHEV isolates from Polish flocks. Sequences from this study are labeled with a star. Phylogenetic trees were generated by the NJ (**a**) and ML (**b**) methods with 1000 bootstrap replicates, as implemented in the MEGA 7.0 software. CL, commercial layer; BB, broiler breeder

The results of serological examination are summarized in Table 1. Anti-aHEV antibodies were detected in 32 (56.1%) out of 57 flocks studied. Of the 1034 serum samples examined, 220 (21.3%) were positive for anti-aHEV antibodies. Half of the broiler breeder flocks were positive for anti-aHEV (23 out of 46 flocks studied), and 25% of the serum samples were positive (197 out of 788). In commercial laying flocks, the seroprevalence was 81.8% (9 out of 11 flocks), while 9.4% of serum samples were anti-aHEV positive (23 out of 246). Seroprevalence was higher in laying hen flocks than in broiler breeder flocks, and birds older than 46 weeks were more likely to be seropositive than those of other age groups (Table 2).

**Table 1:** Results of serological examination of breeder broiler and laying hen flocks

Year	Age of birds (weeks)	No. of flocks	No. (%) of positive flocks	No. of serum samples	No. (%) of positive serum samples	Anti-aHEV Ab titer (min-max)^*^
**Breeder broiler flocks**						
2015	18-58	13	2 (15.4)	201	2 (1.0)	404 – 765
2016	15-54	7	7 (100.0)	183	76 (41.0)	758 – 4507
2017	21-76	26	14 (53.9)	404	119 (29.4)	696 – 4188
**Total:**		**46**	**23 (50.0)**	**788**	**197 (25.0)**	
**Laying hen flocks**						
2015	24-29	5	4 (80.0)	113	15 (13.3)	484 – 810
2017	15-34	6	5 (83.3)	133	8 (6.0)	500 – 6646
**Total:**		**11**	**9 (81.8)**	**246**	**23 (9.4)**	

^*^ The antibody status was considered positive if the titer was 391 or higher

**Table 2:** Results of serological examination of chickens according to age

Age of birds (weeks)	No. of flocks	No. (%) of positive flocks	No. of serum samples	No. (%) of positive serum samples	Anti-aHEV Ab titer (min-max)^*^
≤ 25	17	13 (76.5)	337	39 (12.6)	404 – 2765
26 – 46	20	8 (40.0)	353	38 (10.8)	410 – 6646
≥ 47	20	11 (55.0)	323	128 (39.6)	400 – 4507

^*^ The antibody status was considered positive if the titer was 391 or higher

An RT-PCR assay targeting a portion of the ORF1 gene revealed the presence of specific aHEV RNA in 18 out of 65 tissues sampled (27.7%). The detection rate for liver samples was higher than for spleen samples (16 out of 18 for liver and 8 out of 18 for spleen - Supplementary Figure 1A). Sequencing of the PCR products for ORF1 and ORF2 resulted in 17 and 10 sequences, respectively, that were of sufficient quality to allow phylogenetic analysis. The sequences were submitted to GenBank under accession numbers MH636883-MH636899 for ORF1 fragments and MH6369000-MH636909 for ORF2. Phylogenetic analysis of the partial ORF1 gene showed that the sequences obtained in this study from Polish flocks belonged to genotype 2 (Fig. 1a). The Polish sequences formed three separate clusters. BLASTn analysis of the partial ORF1 gene sequence showed 83-85% nucleotide sequence identity to other aHEV ORF1 sequences in the GenBank database. Phylogenetic analysis of the partial ORF2 gene sequence showed that the sequences from this study formed two main clusters within genotype 2, with an outlier, 18-PL-243, which clustered with the Taiwan genotype 4 KF511797.1 sequence. BLASTn analysis of the partial ORF2 gene sequence showed 85-95% nucleotide sequence identity to other aHEV ORF2 capsid sequences in the GenBank database. There was no correlation between nt sequence and type of production (Fig. 1). All of the isolated sequences originated from different farms, with the exception of isolates 18-PL513 and 18-PL514, which came from one farm (same date of sample collection) but were from two different broiler breeder flocks of 32 and 28 weeks of age, respectively.

The seroprevalence of aHEV in Poland (56.1%) is generally lower than that observed in other countries. Anti-aHEV antibodies were detected in 71%, 57% and 89.7% of flocks examined in the USA, Korea and Spain, respectively [9, 13, 14]. In Taiwan, aHEV antibodies were present in 95.08% of breeder and layer flocks, while a smaller study from China in which only 10 flocks were tested, indicated a seroprevalence of 35.9% [10, 15]. In our study, the seroprevalence in breeder broiler flocks was lower than in layer hens, which might be due to higher biosecurity on breeder broiler farms. The largest number of positive flocks was detected in young birds (≤ 25 weeks of age), but the largest number of positive sera (more positive birds in an infected flock) was detected in older birds (≥ 47 week of age), which is in agreement with studies performed by other authors [12, 13]. The large difference between the detection rates in laying hen flocks at the flock level (81.8%) compared to the bird level (9.4%) can be explained by the fact that all of the laying flocks that were tested were battery-caged birds. It is likely that the virus does not spread as efficiently in a cage system as in broiler breeder flocks, which are not kept in cages.

Phylogenetic analysis revealed that partial ORF1 sequences of isolates from this study clustered with known genotype 2 sequences. The ORF1 sequences formed three clusters in the phylogenetic tree (Fig. 1a). The geographical distribution of those sequences with cluster discrimination is depicted in Supplementary Figure 1b. The partial ORF2 sequences also clustered with genotype 2 sequences, with the exception of the 18-PL243 isolate, which clustered with genotypes 1 and 4. The genotype distinction is made on the whole genome sequence of aHEV, [8, 10]. However, the genotype distinction is not yet recognised by the ICTV, and there is less nt sequence variability between the proposed avian HEV genotypes in comparison to mammalian HEVs [5]. Because the ORF1 sequence of 18-PL243 clustered within genotype 2, we presume that it belongs to aHEV genotype 2, but this can only be confirmed by whole-genome sequencing. All previous partial aHEV sequences from Poland – ORF1 sequences of isolated from 2007 and 2013 as well as ORF2 sequences of isolates from 2012 – belong to genotype 3 (Fig. 1) [4, 16]. In this study, we did not obtain any aHEV sequences belonging to genotype 3. However, the previously described sequences originated from a few individual broiler breeder farms with BLS. It is possible that aHEV genotype 3 is still endemic in some areas or that genotype 2 aHEV has spread more effectively in Poland in recent years and superseded genotype 3. Until now, the genotype 2 has only been detected in the USA and Spain, and genotype 3 was known to be present in locations in or near Poland [4, 13]. In this study, we observed that two partial ORF1 sequences originating from samples obtained from two different breeder layer flocks from the same farm belonged to two different clusters in the phylogenetic tree (Fig. 1a). The nucleotide and amino acid sequence identity between sequences 18-PL513 and 18-PL514 is only 79.6% and 93.2%, respectively. This observation shows that different strains can be present on one farm, which suggests multiple sources of aHEV infection rather than evolution of the virus. In one study performed on a breeder layer farm, it was shown that the virus sequence was stable for approximate 2 years [16]. Recently, genotype 1 and 3 aHEV sequences have been detected in wild birds in Europe [17]. Some of the sequences show high genetic similarity to chicken isolates, suggesting cross-species transmission.

Despite many attempts, we had difficulties in obtaining enough of the ORF2 PCR product for sequencing from some samples. In the past, most PCR reactions to detect and sequence aHEV have been performed either on the helicase gene (ORF1), which is more conserved among aHEV isolates, or the capsid gene ORF2 [14, 18]. In a few studies where both regions were sequenced, there was also more success in obtaining ORF1 sequences. For instance, seven ORF1 sequences and four ORF 2 sequences were obtained in an investigation in Spain [13]. This might be explained by a higher degree of variation within the ORF2 gene or a lower affinity of the primers used. The use of next-generation sequencing techniques should overcome this problem in the future.

In recent years, BLS has appeared to be an emerging disease in Poland, based on verbal information from veterinarians and samples submitted for diagnostic aHEV serology. This coincides with elevated demand for broiler production in Poland, which, since 2014, has become a leading broiler producer in the EU. This study, for the first time, addresses the seroprevalence in broiler breeder and commercial layer flocks in Poland, which is slightly lower than in other countries. In summary, aHEV is widespread in Poland, and genotype 2 is the main type of aHEV circulating in chickens in this country. Moreover, genotype 2 was isolated for the first time in this part of Europe. Further studies are needed to assess the pathogenicity of Polish aHEV strains.

## Electronic supplementary material

Below is the link to the electronic supplementary material.

Supplementary material 1 (PNG 30313 kb)
