# Supplementary figures and images for: Avian hepatitis E virus is widespread among chickens in Poland and belongs to genotype 2
Source: Arch Virol. 2018 Nov 3;164(2):595–9. doi: 10.1007/s00705-018-4089-y (PMC6373257; doi:10.1007/s00705-018-4089-y)

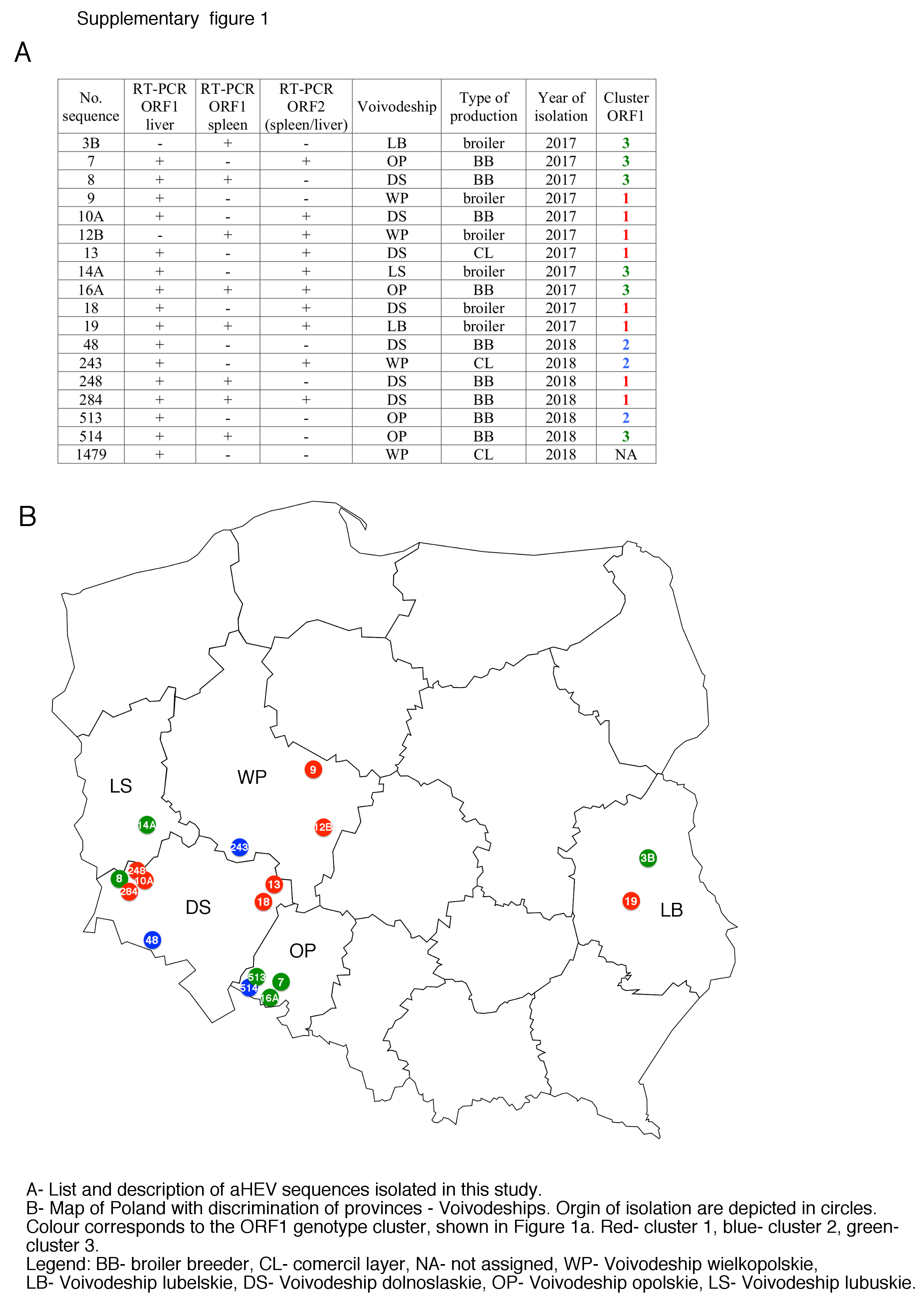

Supplement: Supplementary file 1 — Supplementary material 1 (PNG 30313 kb) [file 705_2018_4089_MOESM1_ESM.png]
